# Evaluation of copy number variation and gene expression in neurofibromatosis type-1-associated malignant peripheral nerve sheath tumours

**DOI:** 10.1186/s40246-015-0025-3

**Published:** 2015-02-15

**Authors:** Laura E Thomas, Jincy Winston, Ellie Rad, Matthew Mort, Kayleigh M Dodd, Andrew R Tee, Fionnuala McDyer, Stephen Moore, David N Cooper, Meena Upadhyaya

**Affiliations:** Institute of Medical Genetics, School of Medicine, Cardiff University, Cardiff, CF14 4XN UK; Almac Diagnostics, 19 Seagoe Industrial Estate, Craigavon, Northern Ireland BT63 5QD UK

**Keywords:** NF1, MPNSTs, Copy number alterations, Gene expression profiling, *SPP1*

## Abstract

**Background:**

Neurofibromatosis type-1 (NF1) is a complex neurogenetic disorder characterised by the development of benign and malignant tumours of the peripheral nerve sheath (MPNSTs). Whilst biallelic *NF1* gene inactivation contributes to benign tumour formation, additional cellular changes in gene structure and/or expression are required to induce malignant transformation. Although few molecular profiling studies have been performed on the process of progression of pre-existing plexiform neurofibromas to MPNSTs, the integrated analysis of copy number alterations (CNAs) and gene expression is likely to be key to understanding the molecular mechanisms underlying NF1-MPNST tumorigenesis. In a pilot study, we employed this approach to identify genes differentially expressed between benign and malignant NF1 tumours.

**Results:**

*SPP1* (osteopontin) was the most differentially expressed gene (85-fold increase in expression), compared to benign plexiform neurofibromas. Short hairpin RNA (shRNA) knockdown of *SPP1* in NF1-MPNST cells reduced tumour spheroid size, wound healing and invasion in four different MPNST cell lines. Seventy-six genes were found to exhibit concordance between CNA and gene expression level.

**Conclusions:**

Pathway analysis of these genes suggested that glutathione metabolism and Wnt signalling may be specifically involved in NF1-MPNST development. *SPP1* is associated with malignant transformation in NF1-associated MPNSTs and could prove to be an important target for therapeutic intervention.

**Electronic supplementary material:**

The online version of this article (doi:10.1186/s40246-015-0025-3) contains supplementary material, which is available to authorized users.

## Background

Neurofibromatosis type 1 (NF1) (MIM# 162200), a complex autosomal dominant disorder with a highly variable clinical phenotype, affects approximately 1 in 4,000 people worldwide (Huson, 2008). Malignant complications are relatively rare manifestations of this disorder but include brain tumours, optic gliomas and malignant peripheral nerve sheath tumours (MPNSTs) [1,2]. MPNSTs can either occur sporadically or instead may develop from the malignant transformation of pre-existing NF1-associated plexiform neurofibromas (PNF) or from a focal subcutaneous neurofibroma. The lifetime risk of developing MPNSTs in NF1 patients is 10%–15% [3,4].

MPNSTs are a significant cause of morbidity and mortality in NF1. Upon first presentation, MPNSTs are often non-resectable because the tumour is already at a late stage of development; metastases may also be present, most commonly in the lung but also in the liver and brain. Consequently, the 5-year survival rate for individuals with these tumours is only 20%–50%, and the 10-year survival rate is even lower (7.5%) [5]. Currently, there are no effective treatments for MPNSTs; although complete surgical excision with clear margins is the therapy of choice, chemotherapy has sometimes also been employed despite its limited effectiveness. Several risk factors confer an increased likelihood of MPNST development, e.g. multiple internal PNFs [6], previous radiation therapy, the presence of an inherited gross genomic deletion that removes the entire *NF1* gene [7], the presence of neurofibromatous neuropathy [8] or a family history of NF1-MPNSTs [9]. Since there are no reliable predictive or prognostic biomarkers for MPNSTs, the progression of a pre-existing plexiform neurofibroma to an MPNST cannot currently be predicted in advance on the basis of molecular (copy number and/or gene expression) profiling despite recent significant advances in this field. Beert et al. (2011) demonstrated recurrent homozygous loss of the *CDKN2A* locus in 15/16 atypical neurofibromas; this finding supports the notion that atypical neurofibromas constitute intermediates between benign neurofibroma and MPNSTs and further suggests that *CDKN2A* loss is an early step in the progression of neurofibroma to MPNSTs [10]. A detailed knowledge of the *NF1* somatic mutational spectrum of MPNSTs is, however, a prerequisite for the development of targeted therapies. Although somatic inactivation of the wild-type *NF1* allele is presumed to be the key step in NF1-associated tumour development, this cannot on its own explain the malignant transformation of benign plexiform neurofibromas to MPNSTs. This indicates that additional genetic (and potentially epigenetic) alterations are required to bring about malignancy.

High-throughput whole genome microarray profiling has proved to be one of the most effective methods to analyse large numbers of clinical samples across multiple tumour types. Indeed, a considerable number of studies have identified somatic copy number alterations (CNA) and concomitant gene expression changes in benign and malignant NF1-associated tumours [11-30]. However, despite the plethora of previous array-based investigations, relatively few genes have so far been identified that are consistently mutated across multiple MPNSTs. Such frequently mutated genes may harbour ‘driver mutations’ (as opposed to ‘passenger mutations’) that promote NF1 malignant transformation. MPNST development is clearly a complex multistep process in which the mutation of a large number of genes, contributing to the deregulation of multiple signalling and regulatory pathways, is to be expected. Whole genome analysis and, in particular, the focussed investigation of specific pathways are likely to be key to determining the underlying molecular mechanisms involved in MPNST tumorigenesis. Genes that could be informative in a prognostic context may be identified through the use of integrated CNA/gene expression analysis performed on the same sample sets [31,32]. To this end, the present study employed Affymetrix Human Exon 1.0 ST Arrays to screen both PNF and MPNST tumour DNAs from nine unrelated NF1 patients to generate data on differences in gene expression at the whole genome level between benign and malignant tumours with which to compare with previously generated CNA data derived from the same tumours in order to disclose novel genes and pathways that could be important in MPNST development [25].

## Results

### Identification of differentially expressed genes between neurofibromas and MPNSTs

**Figure 1:**
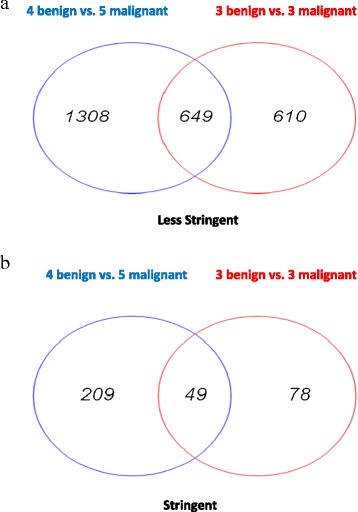
**Venn diagram comparison based upon the less stringent and stringent gene list. a**. Venn diagram based upon the less stringent gene lists from the 4 vs. 5 and 3 vs. 3 pairwise comparisons. **b**. Venn diagram based upon the stringent gene lists from the 4 vs. 5 and 3 vs. 3 pairwise comparisons.

For differential expression analysis, different sets of genes were identified from the various analyses (Figure 1a,b) that we performed: (1) 4 benign tumour samples vs. 5 malignant tumour samples with stringent criteria applied, (2) 4 benign tumour samples vs. 5 malignant tumour samples under less stringent criteria, (3) 3 benign tumour samples vs. 3 malignant tumour samples under stringent criteria and (4) 3 benign tumour samples vs. 3 malignant tumour samples under less stringent criteria (Additional file 1: Table S2). The genes identified employing the less stringent criteria from the 4 vs. 5 analyses were cross-compared with the corresponding list of genes identified by means of the 3 vs. 3 analysis (Figure 1a). The same process was then followed for those genes identified using the more stringent criteria (Figure 1b). Given the relatively small number of patients included in this study, the presence of outliers (patients exhibiting variable expression compared with the median) was expected to have a considerable influence on the combined results. With this in mind, the analysis as described above was performed with and without those outlier/variable samples.

The number of genes identified as being differentially expressed between benign and malignant tumours under more or less stringent criteria in the 3 vs. 3 and 4 vs. 5 analyses can be found in Table 1. Under the less stringent criteria, statistical testing employed a *P* value threshold of 0.05, which served to identify genes displaying ≥1.3-fold (absolute) differential gene expression. In the differential analysis, which considered all profiled samples (4 benign vs. 5 malignant), a total of 1,957 genes were identified as being differentially expressed between benign and malignant tumours whereas in the differential analysis which excluded the three samples at the expression range extremes (3 benign vs. 3 malignant), only 1,259 genes were identified. The two different conditions of stringency were used for the sake of comparison. However, for the final analysis, the genes identified under the more stringent criteria were used. Under the more stringent criteria, statistical testing employed a *P* value threshold of 0.005, which was deemed to be sufficient to identify genes displaying 1.5-fold or more (absolute) differential gene expression. In the 4 vs. 5 stringent gene list analysis, 258 genes were identified as being differentially expressed between benign and malignant tumours whereas in the 3 vs. 3 analyses, the number of genes identified was 127. The top 20 significantly differentially expressed genes identified using the more stringent criteria are given in Table 2.

**Table 1:** **Definitions of stringent and less stringent analytical criteria and the numbers of genes detected by the 3 vs. 3 and 4 vs. 5 analyses under both sets of criteria**

	**Less stringent criteria**	**Stringent criteria**
Statistical testing *P* value	0.05	0.005
Fold change	1.3	1.5
Number of genes 4 vs. 5	1,957	258
Number of genes 3 vs. 3	1,259	127

Definition of stringent and less stringent analytical criteria and the numbers of genes detected by the t vs. 3 and 4 vs. 5 analyses under both sets of criteria.

**Table 2:** **The top 20 genes identified as being significantly differentially expressed between MPNSTs and PNFs in the more stringent analysis of 3 vs. 3 samples**

**Transcript ID**	**Gene symbol**	**Ref seq**	**Mean expression**	**Mean expression**	**Fold change**	***P*** **value**	**Chromosome**	**Start**	**Stop**
			**Benign**	**Malignant**	**Benign vs. malignant**	**Benign vs. malignant**			
2584134	*FAP*	NM_004460	4.87	8.75	14.75	0.0001	2	163027200	163101589
4013828	*NSBP1*	NM_030763	5.22	3.67	−2.92	0.0001	X	80211860	80457431
3917555	*KRTAP13n4*	NM_181600	3.77	3.13	−1.55	0.0001	21	31797943	31809269
3908934	*PTGIS*	NM_000961	4.58	6.60	4.06	0.0002	20	48120414	48185550
2764192	*KIAA0746*	NM_015187	4.33	5.82	2.80	0.0002	4	25722248	25865344
2735027	*SPP1*	NM_001040058	5.65	12.06	85.19	0.0002	4	88408243	88904561
3532353	*FAM177A1*	NM_001079519	8.01	7.11	−1.86	0.0003	14	35508201	35582327
2779231	*ADH1B*	NM_000668	6.50	3.18	−9.99	0.0003	4	100226131	100242895
2406926	*GRIK3*	NM_000831	7.20	3.85	−10.19	0.0004	1	37266614	37524753
3916290	*FLJ42200*	AK124194	3.32	4.05	1.65	0.0004	21	25353415	25920256
2970942	*COL10A1*	NM_000493	3.56	4.67	2.17	0.0004	6	116440092	116518549
2450865	*CSRP1*	NM_004078	9.09	7.28	−3.50	0.0005	1	201445919	201481500
2918982	*GRIK2*	NM_175768	7.02	3.47	−11.66	0.0005	6	101841683	102690448
3204648	*CD72*	NM_001782	4.08	5.08	1.99	0.0005	9	35609970	35646790
4006326	*EFHC2*	NM_025184	4.84	3.42	−2.67	0.0005	X	44006888	44309044
2586845	*SLC25A12*	NM_003705	5.56	6.30	1.68	0.0006	2	172639117	172750960
2579439	*GTDC1*	NM_001006636	6.95	5.92	−2.05	0.0006	2	144702190	145123135
3872053	*PEG3*	NM_006210	5.58	4.17	−2.65	0.0006	19	57285930	57352082
2801526	*CCT5*	NM_012073	8.62	9.33	1.64	0.0006	5	10250111	10266501
3548152	*TDP1*	NM_018319	4.17	5.49	2.50	0.0007	14	90421293	90511103

The top 20 genes identified as being significantly differentially expressed between MPNSTs and PNFs in the more stringent analysis of 3 vs. 3 samples.

The *SPP1* gene [NCBI: NM_001040058] (osteopontin; OPN) was found to exhibit the most significantly elevated differential mean expression level between the benign and malignant tumours. *SPP1* was therefore selected for further functional analysis to assess its possible role in malignant transformation. Exon array analysis identified a mean expression level of 12.06 (log2) for *SPP1* in malignant tumours and a mean expression level of 5.65 (log2) in benign plexiform neurofibromas. This equates to an 85-fold increase (based on anti-logged LS means) in expression in MPNSTs compared to benign plexiform neurofibromas (*P* = 0.0002) (Table 2). Since this was the highest fold change in expression observed between benign and malignant tumours for all the genes identified in this analysis, an association with malignant transformation was strongly suspected. Significant reductions in the expression of *ADH1B* and *GRIK2* (*P* values 0.0003 and 0.0004, respectively) in MPNSTs as compared to benign plexiform neurofibromas were however also observed (Table 2).

### Quantitative real-time PCR (q-PCR)

Gene expression changes initially detected by Affymetrix Human Exon 1.0 ST Array analysis were validated by q-PCR. We aimed to determine where there was a correlation between the presence of a copy number alteration and gene expression (either a concomitant increase in copy number and gene expression or a decrease in both) as assessed by quantitative PCR. This was determined by identifying whether the same directional change as noted above was identified in the array data from the current study and CNA data from the previous analyses [25] (Additional file 2: Table S3). A correlation was noted for 11 of the 20 differentially expressed genes analysed (Additional file 3: Figure S1) (*ADH1B*, *FAP*, *FLJ42200*, *GRIK2*, *GTDC1*, *KIAA0746*, *KRTAP13-4*, *PEG3*, *NSBP1, PTGIS* and *SPP1*). For six of the genes (*CCT5, CD72, COL10A1, FAM177A1, SLC25A12* and *TDP1*), we were unable to identify a correlation (i.e. concordance) between the copy number alteration and gene expression in six of the tumour samples tested. For the remaining three genes (*CSRP1, GRIK3* and *EFHC2*), half the samples yielded findings compatible with, and anticipated from, the results of the exon array experiments; the remaining samples either demonstrated no change in gene expression or the opposite change to that anticipated on the basis of the exon array results (i.e. a decrease in gene expression identified by array, increase in gene expression identified by Q-PCR) (see Additional file 3: Figure S1).

### Integration of copy number analysis with differentially expressed genes

**Figure 2:**
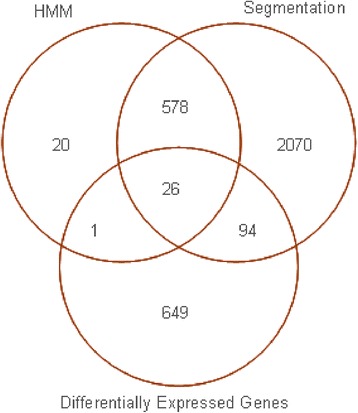
**Venn diagram comparing the numbers of genes from the HMM and segmentation copy number analyses with the genes found to be differentially expressed.**

CNA data were derived from a previously published analysis of the same tumours [25]. In total, 27 genes were common to HMM and expression analysis, whereas 120 genes were common to segmentation and expression analysis. We then selected those genes that were common to expression analysis and HMM and/or segmentation analysis (121 genes in total; see Figure 2). Of these 121 genes, gene expression and CNA data were concordant in 76 (63%) cases, with all 76 genes displaying the same directional change of both CNA and gene expression (Additional file 2: Table S3). The 76 genes that were concordant for copy number and gene expression changes were regarded as potential candidates for involvement in malignancy (Additional file 2: Table S3).

### Pathway analysis

The 76 concordant genes, which were common to both the CNA and expression analyses, were interrogated using all pathways in the METACORE database. Pathways in which more than one gene was subject to concomitant alterations in both copy number and expression were then listed. The top ten pathways (by *P* value) are given in Table 3. These pathways include the glutathione metabolism, Wnt signalling, cell adhesion and alpha-6/beta 4 integrin pathways.

**Table 3:** **Pathway Enrichment analysis**

	**Pathway name**	***P*** **value**	**Genes**	**Fold change (3 benign vs. 3 malignant)**	**Fold change (4 benign vs. 5 malignant**
1	Glutathione metabolism	0.00056	*GSTM1*	−3.25	−2.27
			*GSTM2*	−3.25	−2.27
			*GSTM4*	−3.25	−2.27
2	Glutathione metabolism/human version	0.00059	*GSTM1*	−3.25	−2.27
			*GSTM2*	−3.25	−2.27
			*GSTM4*	−3.25	−2.27
3	Glutathione metabolism/rodent version	0.00073	*GSTM1*	−3.25	−2.27
			*GSTM2*	−3.25	−2.27
			*GSTM4*	−3.25	−2.27
4	Development: Wnt signalling pathway. Degradation of beta-catenin in the absence Wnt signalling	0.00106	*CSNK1D*	2.42	1.99
			*DAB2*	3.46	2.65
5	Cell adhesion: PLAU signalling	0.00445	*HGF*	7.80	10.97
			*SERPINE1*	5.74	4.54
6	Role of alpha-6/beta-4 integrins in carcinoma progression	0.00589	*HGF*	7.80	10.97
			*LIMK1*	2.04	2.23
7	Development: TGF-beta-dependent induction of EMT via MAPK	0.00641	*DAB2*	3.46	2.65
			*SERPINE1*	5.74	4.54
8	Transport: macropinocytosis regulation by growth factors	0.01130	*HGF*	7.80	10.97
			*LIMK1*	2.04	2.23
9	Development: regulation of epithelial-to-mesenchymal transition (EMT)	0.01165	*HGF*	7.80	10.97
			*SERPINE1*	5.74	4.54
10	Transport: clathrin-coated vesicle cycle	0.01421	*PREB*	1.45	1.43
			*DAB2*	3.46	2.65

The pathways were ranked by hypergeometric *P* values, and a summary of the top ten pathways is reported. The *P* value represents the probability that a gene set of this size would co-occur by chance alone. Network objects represent the ratio of the number of genes from the list compared to the total number of genes known to be associated with the pathway.

### SPP1 knockdown in multiple MPNST cell lines impairs tumour formation, wound healing and invasion

**Figure 3:**
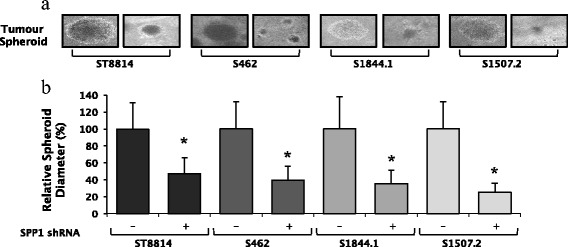
***SPP1*** **knockdown reduces tumour size in soft agar.** Stable ST8814, S462, S1844.1 and S1507.2 cell lines, expressing either non-target or *SPP1* shRNA as indicated, were subjected to tumour spheroid growth assays. **a)** Photographs of 40 tumours were taken after 2 weeks of incubation. The diameter of all tumours was measured using ImageJ software; the scale bar on images represents 250 μm. **b)** The percentage of tumour size was calculated from the 40 tumours for both the control and *SPP1* shRNA. *SPP1* knockdown significantly reduced tumour size in all four MPNST cell lines, consistent with a role for *SPP1* in tumour growth (*P* = 0.0001), *P < 0.05 when comparing treated vs. untreated cells.

**Figure 4:**
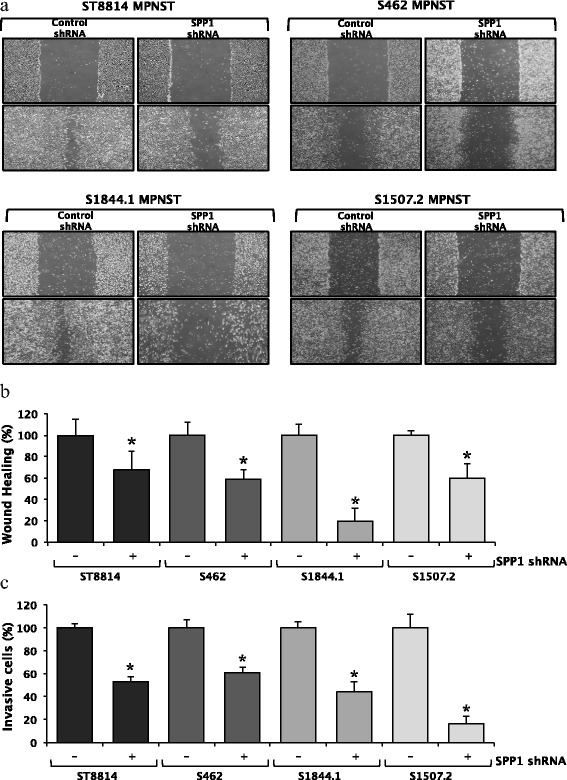
***SPP1*** **knockdown impairs wound healing and cell invasion.** Stable ST8814, S462, S1844.1 and S1507.2 cell lines, expressing either non-target or *SPP1* shRNA, as indicated, were subjected to a wound healing assay. **a)** Pictures of cells were taken at 0 and 18 h after wounding. **b)** The percentage of cell migration was calculated using three individual experiments. *SPP1* knockdown significantly reduced cell migration during wound healing in all four MPNST cell lines [*P* values for the four cell lines were as follows: ST8814 (0.03), S462 (0.001), S1844.1 (0.001), S1507.2 (0.003)]. **c)** *SPP1* knockdown significantly reduced cell invasiveness; crystal violet was used to stain invasive cells. Cells were then eluted with 1% (w/v) SDS, and the absorbance was read at 550 nm on a Genova MK3 Lifescience Analyser; the number of cells was calculated by plotting the absorbance against a standard curve. The percentage of invasive cells was calculated in three individual experiments. *SPP1* knockdown significantly reduced cell invasion in all four MPNST cell lines, suggesting a potential role for *SPP1* in metastasis [*P* values for the four cell lines were as follows: ST8814 (0.0001), S462 (0.001), S1844.1 (0.0001), S1507.2 (0.001). *P < 0.05 when comparing treated vs. untreated cells].

**Figure 5:**
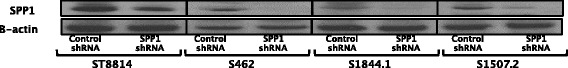
**To confirm efficient knockdown, SPP1 protein levels were compared between control and** ***SPP1*** **knockdown cell lines by Western blot.** β-actin was used as a loading control. SPP1 protein levels were significantly reduced after SPP1 knockdown in all MPNST cell lines.

Given that we observed significant differences in the expression level of *SPP1* in MPNSTs as compared with the benign plexiform neurofibromas and the fact that *SPP1* plays significant roles in both tumorigenesis and metastasis [33], we knocked down expression of the *SPP1* gene in four MPNST cell lines (ST8814, S462, S1844.1 and S1507.2) and examined both the ability of these cell lines to form tumours (Figure 3a) and the effect of knockdown on wound healing and invasion. Both control and *SPP1* knockdown cell lines formed tumour colonies in soft agar. However, *SPP1* knockdown caused a significant reduction in tumour spheroid size (*n* = 40, *P* = 0.0001) in all MPNST cell lines tested (Figure 3b). We next analysed the effects of *SPP1* knockdown on cell migration during wound healing (Figure 4a). In all cell lines, knockdown of *SPP1* robustly inhibited wound closure (Figure 4b) (*P* values for the ST8814, S462, S1844.1 and S1507.2 cell line were 0.03, 0.001, 0.001 and 0.003, respectively), suggesting a possible role for *SPP1* in metastasis. This role was further supported by the results of cellular invasion assays, which revealed that *SPP1* knockdown significantly reduces the cells’ invasive properties (*P* values for the ST8814, S462, S1844.1 and S1507.2 cell line were 0.0001, 0.001, 0.0001 and 0.001, respectively) (Figure 4b,c). Validation of specific gene knockdown using short hairpin RNA (shRNA) clones was completed by Western blot analysis. A β-actin control blot was performed to confirm that there were no shRNA off-target effects (Figure 5). In summary, *SPP1* knockdown with shRNA resulted in a significant reduction in both wound healing and invasiveness.

## Discussion

The functional loss of neurofibromin, due to *NF1* gene inactivation, leads to increased cell growth and proliferation through constitutive Ras pathway signalling. Although this is necessary for benign neurofibroma formation, it is insufficient to explain the malignant transformation of a benign PNF to an MPNST, since additional genetic lesions (and/or epigenetic modifications) are required for this to occur [25].

Initially, this study aimed to identify genes that contribute to the malignant transformation of benign neurofibromas by establishing which genes were differentially expressed between benign and malignant tumours in NF1 patients. A previously compiled list of genes located within regions encompassed by copy number alterations (CNAs) identified in the NF1-MPNSTs which were also used in the current study [25] was then cross-compared with a list of genes differentially expressed in the same NF1-MPNST samples, which were analysed for this study through Affymetrix exon array analysis. Finally, an additional study involving pathway analysis was performed on those genes that were common to the results of the CNA and exon array analyses.

In previous studies of NF1-MPNSTs, many genetic alterations, including copy number alterations and differential gene expression, have been identified but no specific molecular signature pathognomonic of malignant transformation has so far been defined [11-30]. Identifying genes that are differentially expressed between benign and malignant tumours not only promises to improve our understanding of the process of malignant transformation but should also aid in identifying potential therapeutic targets.

This study employed the GeneChip Human Gene 1.0 ST array (Affymetrix, Santa Clara, USA). This array is a whole transcript-based array for gene expression profiling which, unlike older Affymetrix arrays, queries the entire transcript instead of just the 3′ end [34]. Additionally, this array uses a labelling protocol that generates biotinylated sense strand DNA instead of complementary RNA (cRNA), thereby yielding DNA-DNA duplexes, which are more specific than the RNA-DNA duplexes generated using standard protocols [35]. This array provides approximately 25-mer probes designed to be distributed across the transcribed regions of each gene with a median of 26 probes per gene, giving two complementary levels of expression analysis in a single experiment, both “exon-level” and “gene-level” analysis. The array interrogates 28,869 well-annotated genes with 764,885 distinct probes. The array is based on the March 2006 (UCSChg18, NCBI Build 36) human genome sequence assembly. The Affymetrix Human Exon 1.0 ST array contains 17,881 transcripts. Whole genome Affymetrix exon arrays have been employed in a number of different studies of cancer including non-small cell lung, colon, bladder and prostate cancer [36,37].

The comparative transcriptome analysis reported here identified the *SPP1* gene to be the single most differentially overexpressed gene in NF1-MPNSTs as compared to benign tumours. *SPP1* was selected for further study not only for this reason but also because of its well-documented involvement in cell signalling, tumorigenesis and metastasis [38-49]. shRNA knockdown of four different MPNST cell lines revealed a significant reduction in tumour colony size growth, wound healing and cell invasion, thereby supporting a role for the increased expression of *SPP1* in the malignant transformation and invasion of cells during NF1-MPNST development. *SPP1* is an extracellular matrix protein with cytokine properties. It is involved in extracellular matrix (ECM) and adhesion-related pathways where it performs key roles in cell-cell communication, focal adhesion, immune cell activation and immune cell migration. It plays an essential role in the pathway that leads to type I immunity, thereby enhancing the production of interferon-gamma and interleukin-12 and reducing interleukin-1 synthesis. In terms of an association with cancer, *SPP1* has been shown to promote the growth of different tumours [38-49]. Cells harbouring activated RAS have been found to exhibit a higher level of *SPP1* [50]. It could therefore be that the activation of RAS through the functional loss of neurofibromin (due to *NF1* gene inactivation) gives rise to the overexpression of *SPP1*. Furthermore, the use of *SPP1* inhibitors such as agelastatin A has successfully reduced colony formation, migration and invasion in human breast cancer cell lines [51]. Whilst *SPP1* has the highest level of differential gene expression, the remaining genes in Table 2 would warrant further exploration in future studies. This would help to explore further the relationship with NF1 of not only SPP1 but the other identified statistically significant genes, which are differentially expressed between benign and malignant NF1 tumours.

In an attempt to explore the relationship between gene expression and copy number variation in the context of NF1 tumorigenesis, we attempted to integrate previous copy number data derived from the MPNSTs under study here [25], with the newly generated data on differentially expressed genes. The same tumour samples were utilised for both analyses. The identification of prognostic biomarkers for NF1-MPNSTs using gene expression microarrays is challenging in that there are very few candidate genes in common between the different studies so far performed [11-30]. However, over the last 5 years, studies of cancer have begun to integrate gene expression and copy number analysis in an attempt to explore underlying mechanisms of tumorigenesis and to identify potential gene and pathway biomarkers [31,32]. Such an integrative approach has led to advances in our understanding of the role of copy number alterations in tumorigenesis. Thus, for example, the Cancer Genome Atlas project [52] is generating multiple datasets using different platforms (e.g. gene expression and copy number analysis) from the same set of patients. Although the increased expression of a particular gene does not by itself constitute direct evidence for the role of that gene in tumorigenesis, concomitant copy number alterations may serve to disrupt metabolic and physiological processes, thereby contributing indirectly to tumorigenesis [31,32]. There is however compelling evidence for a *cis*-dosage effect of CNA on gene expression [53], and this relationship can facilitate the identification of novel genes involved in tumorigenesis as well as other aspects of cancer biology. In any such analysis, it is important to be aware of cellular heterogeneity within the tumour [54].

Work on different tumours, including breast, lung, prostate, hepatocellular carcinoma and melanoma, has yielded an estimate of the proportion of all differentially expressed genes whose expression is concordant with their copy number status. This has been shown to vary between 32% and 78% [31,32,55,56]. In this context, it is encouraging that in the current study, 63% of genes were found to be concordant in terms of their expression and copy number status. However, although *SPP1* was found to be the most differentially expressed gene in the initial part of this study, we failed to find any copy number changes in MPNSTs in our earlier study and thus its expression was not concordant with the copy number changes [25]. For this reason, we propose that the observed upregulation of *SPP1* gene expression may not have been modulated by a copy number alteration. This is especially in view of the fact that no copy number alterations involving *SPP1* have previously been reported in a variety of cancers and tissue types (by reference to COSMIC and CONAN) [57,58], including NF1 MPNSTs [40]. Thus, in the absence of gene duplication/amplification/deletion, it may be that there are other genetic mechanisms including DNA methylation, point mutations, up- or downregulated transcription factors, regulation of messenger RNA (mRNA) transcription or microRNAs (miRNAs) [59-61] that could influence the expression of the non-concordant genes (37%) identified in this study.

Finally, we performed pathway analysis on the 76 genes that were common to the copy number array and exon array data. Integration of clinical information with copy number and gene expression data has led to the identification of genes common to CNA regions and expression array datasets that are consistently associated with clinical outcomes including lung cancer, thereby underlining the clinical relevance and utility of such data sets [56]. The top ten statistically significant pathways were noted from those where two or more genes exhibiting alterations in copy number and/or expression belonged to the same pathway (Table 3). These pathways included glutathione metabolism, Wnt signalling, cell adhesion PLAU and intracellular signalling by alpha-6/beta-4 (a6b4) integrins. The Wnt signalling pathway, including the genes *CSNK1D* and *DAB2* in this data set (Table 3), is of particular interest. The *APC* gene belongs to the Wnt pathway and is somatically mutated in various cancers and also in familial adenomatous polyposis, which results from inherited *APC* gene mutations [62]. In human colon cancer, SPP1 is a transcriptional target of aberrant Wnt signalling, and *SPP1* expression alone predicts survival [63]. Vinas et al. (2010) demonstrated that the antiapoptotic role of Wnt was mediated by SPP1, a direct Wnt target gene, and SPP1 was reduced by Wnt antibody administration *in vivo* [64]. Using comparative transcriptome analysis, Mo et al. (2013) previously demonstrated that PI3K and β-catenin signalling are involved in the promotion of MPNST growth [26]. Furthermore, using a sleeping beauty forward genetic screen, the Wnt pathway has been found to be a driver of MPNST development [27]. Most interestingly, in a recent study, 20 Wnt genes exhibited altered expression in MPNST biopsies and cell lines in comparison to benign neurofibromas [30]. Taken together, it has become clear that progression to malignancy requires many genomic alterations acting in concert. Importantly, our results appear to concur with the findings of previous studies that the canonical Wnt signalling pathway is likely to be a key driver of MPNST development. Thus, members of the Wnt pathway may not only constitute potential biomarkers of MPNST tumorigenesis but also represent potential therapeutic targets for small molecule inhibitors [26-28].

In addition to the Wnt signalling pathway, other pathways in Table 3 are also thought to be involved in cancer progression. Specifically, although there is no clear association reported with NF1, altered glutathione (GSH) metabolism is thought to be a major mechanism of chemoresistance and GSH levels are reportedly elevated in non-small cell lung cancer [65]. In addition, genetic variations in genes involved in the glutathione and DNA repair pathways are associated with non-small cell lung cancer survival [66]. Further analysis of the pathways in Table 3 would be important to ascertain the role of the *NF1* gene in other cancers.

## Conclusions

Although MPNSTs only develop in approximately 15% of NF1 patients, they represent a frequent cause of lethal progression of the NF1 phenotype. It is clear that many genetic (and potentially epigenetic) factors contribute to abnormal tumour progression in these neoplasms [67,68]. The prognosis for individuals diagnosed with an MPNST is usually very poor, especially as treatment options for MPNSTs are currently very limited and complete surgical excision with clear margins has proved to be very difficult. In knockdown experiments involving shRNA for *SPP1*, we found that cell migration was reduced in four different MPNST cell lines. This exploratory study supports the idea of a direct role for *SPP1* in MPNST development and metastasis. Although obtaining MPNST samples can be challenging, this preliminary study warrants confirmation in a larger panel of samples as the study has a limitation in that it is based on a small cohort of samples. In addition to the well-studied role of osteopontin in other cancers [38-49], the expression of *SPP1* is regulated by Wnt signalling, one of the pathways that we identified as playing a role in the progression of benign plexiform neurofibromas to MPNSTs [69]. This is most encouraging in terms of the potential for those genes and pathways newly identified in this study to help in understanding the molecular basis of tumorigenesis and malignant transformation in NF1 as well as providing targets for therapeutic intervention in NF1-MPNST development.

## Methods

### Patient samples and RNA preparation

DNA and RNA were isolated from the same segment of tumour. RNA was extracted from nine NF1-associated tumours with biallelic *NF1* gene mutations (comprising four benign PNFs and five high grade MPNSTs) from nine unrelated NF1 patients using the TRIZOL method (Invitrogen) as previously described [25]. The same tumour samples were utilised in the current study and the previous analyses for microarray analysis [25] to enable analysis of paired CNA and gene expression data. All total RNA samples were assessed for purity and integrity by means of an Agilent Bioanalyser. Samples were renamed and randomised in order to avoid order bias or batching effects. Analysis of gene expression by Affymetrix Human Exon 1.0 ST Array was performed by Almac Diagnostics (Craigavon, UK). The Affymetrix Human Exon 1.0 ST array contains 17,881 transcripts. Standard operating protocols, as provided by the manufacturer, were used to PCR amplify and hybridise nine NF1-associated tumour-derived cDNA samples on Affymetrix Human Exon 1.0 ST arrays.

### Ethics statement

All patients provided informed consent and the study was approved by the Research Ethics Committee (REC) for Wales and appropriate institutional review boards.

### Identification of differentially expressed genes between benign neurofibromas and MPNSTs

A quality control (QC) assessment of the Exon array profiles was performed to examine standard Affymetrix quality parameters, expression distribution patterns and array profile relationships. Overall, a high quality level was achieved (median percent present = 65.99%), although the average expression level of all probe sets across the array profiles was quite variable. “percent present” represents the proportion of probe sets ‘present’ or detected as defined by the DABG (detection above background) algorithm. The DABG algorithm yields a detection metric (*P* value) generated by comparing perfect match probes to a distribution of background probes. A probe set was considered to be ‘present’ if the DABG *P* value was ≤0.01. Further information is available at www.affymetrix.com. In an attempt to mitigate the variable expression profile observed, differential gene expression analysis was performed with and without those samples from either end of the observed expression range, namely S0342F0011 (malignant), S0342F0020 (malignant) and S0342F0030 (benign). The results that were common to both differential analyses were taken forward for further analysis. Probe-level data were analysed using the robust multi-array average (RMA) method to generate gene-level measurements [70].

The expression levels of all genes on the array were compared between benign PNFs and MPNSTs, at two different levels of stringency. Under the less stringent criteria, statistical testing employed a *P* value threshold of 0.05, which served to identify genes displaying 1.3-fold or more (absolute) differential gene expression. The two levels of stringency that were used as fold change cut-offs, in this sense, were essentially arbitrary but nevertheless provide an element of choice in terms of prioritising targets to follow up. We have filtered at 1.3, which may be interpreted as “low stringency”. However, combined with a significant *P* value, this can often be valid in the context of those biological systems where some changes are expected to be subtle yet functionally relevant. Moreover, although the fold change estimations from microarray data compared to qPCR are frequently underestimated, it is often possible to validate findings by qPCR from the lower fold change space of microarray data. Under high stringency criteria, statistical testing employed a *P* value threshold of 0.005, which was deemed to be sufficient to identify genes displaying 1.5-fold or more (absolute) differential gene expression. This approach allowed us to interrogate two different sets of expression data, thereby minimising the loss of important information.

### Quantitative real-time PCR (q-PCR)

All of the top 20 differentially expressed genes were independently assessed by quantitative PCR using RNA isolated from additional MPNST samples, not used in the previous or current study. Due to the small initial sample size, we felt that it was necessary to determine the validity of the top 20 genes in an independent cohort of samples. These 20 genes, plus the *B2M* [NCBI: NM_004048] endogenous control identified in our analysis were: *FAP* [NCBI: NM_004460], *NSBP1* [NCBI: NM_030763], *KRTAP13-4* [NCBI: NM_181600], *PTGIS* [NCBI: NM_000961], *KIAA0746* [NCBI: NM_015187], *SPP1* [NCBI: NM_001040058], *FAM177A1* [NCBI: NM_001079519], *ADH1B* [NCBI: NM_000668], *GRIK3* [NCBI: NM_000831], *FLJ42200* [NCBI: AK124194], *COL10A1* [NCBI: NM_000493], *CSRP1* [NCBI: NM_004078], *GRIK2* [NCBI: NM_175768], *CD72* [NCBI: NM_001782], *EFHC2* [NCBI: NM_025184], *SLC25A12* [NCBI: NM_003705], *GTDC1* [NCBI: NM_001006636], *PEG3* [NCBI: NM_006210], *CCT5* [NCBI: NM_012073] and *TDP1* [NCBI: NM_018319]. qPCR was performed as previously described [25]. Primers for all genes are listed in Additional file 4: Table S1.

### Cell lines and maintenance

ST8814 MPNST-derived cell lines were purchased from ATCC (distributed by LGC Standards, Middlesex, UK). The S462, S1507.2 and S1488.1 MPNST cell lines were a kind gift from Prof. Mautner (University of Hamburg, Germany) and the late Prof. Guha (University of Toronto, Canada). After *SPP1* knockdown, cell lines were cultured and maintained in Dulbecco’s Modified Eagle Medium (DMEM) supplemented with 10% (v/v) foetal bovine serum (FBS) and 1% (v/v) penicillin-streptomycin and 5 ng/ml puromycin in a humidified incubator (5% CO_2_ at 37°C).

### Lentivirus generation and shRNA knockdown of SPP1

Both *SPP1* shRNA (Clone ID: NM_000582.2-597s1c1) and non-target control MISSION shRNA (Clone ID: SHCO16) in pLKO.1 vector (Sigma-Aldrich Company Ltd., Dorset, UK) were packaged into lentivirus using HEK293T cells co-transfected (lipofectamine 2000, Life Technology, Paisley, UK) with pLP1, pLP2 and pLP (VSVG). Confluent MPNST cell lines were infected with shRNA-containing lentivirus (*SPP1* or non-target control) and selected over 2 weeks with 5 μg/ml puromycin (Life Technology, Paisley, UK). Puromycin-selected mixed cell populations were then used for tumour formation, wound healing and invasion assays.

### Tumour spheroid assays

Two-layered soft agar assays were carried out in six-well plates. MPNST cell lines were plated in complete DMEM media in 0.3% (v/v) agar at a density of 3 × 10^5^cells/mL over a 0.6% (v/v) agar layer. The agar was then overlaid with complete DMEM media supplemented with 0.1 μM puromycin (Life Technology, Paisley, UK). Colonies of MPNSTs were grown for 14 days at 37°C in 5% CO_2_. Media were changed twice a week. Representative pictures were taken using an inverted AMG EVOS microscope equipped with an Olympus camera. The diameter of tumour spheroids was measured using ImageJ software; the chamber of a haemocytometer (250 μm) was used as a scale bar for measurement.

### Wound healing

Cells were seeded in 60-mm plates and left to reach 100% confluency. Cells were then synchronised in 1% (v/v) FBS DMEM for 24 h and “wounded” with a pipette tip. Dead cells were removed with PBS wash and then replaced with DMEM (10% (v/v) FBS). Pictures were taken at 0 and 12–18 h using an inverted AMG EVOS microscope equipped with an Olympus camera.

### Invasion assays

Transwell permeable supports with 6.5-mm diameter inserts, 8.0-μm pore size and a polycarbonate membrane (Corning, cat no: 3428) were used to perform the invasion assays. Cells were grown in a 75-cm^2^ flask with standard medium (10% (v/v) FBS) until confluent. Cells were then harvested using Trypsin-EDTA. Cells were counted using a haemocytometer; 1 × 10^6^ cells were resuspended in DMEM containing 1% (v/v) FBS. The top chamber of the Transwell was filled with 300 μl BD Matrigel basement membrane matrix (1 mg/ml). The Matrigel was incubated at 37°C for 4 h to allow it to gel. Cells were then seeded in the upper chamber of the Transwell; the lower chamber was filled with 600 μl standard culture medium (10% (v/v) FBS) and 5 mg/mL fibronectin (R&D systems, Abingdon, UK), as an adhesive substrate. Cells were incubated at 37°C 5% CO_2_ for 3 days. The proportion of adherent cells was then determined by fixing the cells with methanol and acetone (1:1) for 20 min at −20°C. Cells were then stained with crystal violet (5 mg/ml) in ethanol for 10 min, followed by a stringent wash with dH_2_O until the water ran clear. Crystal violet-stained cells were eluted with 1% (w/v) SDS and the absorbance read at 550 nm on a Genova MK3 Life Science Analyser (Jenway Scientific, Staffordshire, UK). Three replicates were performed per cell line to enable statistical analysis.

### Western blotting

The NuPage Novex gel system was used for electrophoresis as described in the manufacturer’s protocol (Life Technology, Paisley, UK). Protein samples were resolved on 4%–12% Bis-Tris gels to identify a band size of 66 kD. Proteins were then transferred to a polyvinylidene fluoride (PDVF) membrane purchased from Millipore U.K. Ltd. (Watford, UK), blocked in 5% (w/v) dry milk powder in standard Tris-buffer saline supplemented with 0.1% (v/v) Tween [as recommended by Cell Signalling Technology Inc. (Danvers, MA, USA)] for 4 h. Membranes were incubated at 4°C overnight in primary antibody (1:200 dilutions in 2% (w/v) BSA in TBS-T) then washed twice for 4 min in TBS-T and incubated in the appropriate HRP-conjugated secondary antibody (1:10,000 dilution in 5% (w/v) Marvel in TBS-T) for a minimum of 30 min at room temperature. Membranes were washed four times for 3 min with TBS-T and then incubated in enhanced chemiluminescence (ECL) solution (GE Healthcare Life Sciences, Buckinghamshire, UK) for 1 min. Konica Medical Film was used to visualise the signal, and the exposed films were developed using a Konica Minolta SRX-101A developer.

### Statistical analysis

SPSS software was used for the statistical analysis of tumour spheroid formation (*n* = 40) and the wound healing (*n* = 3) and invasion (*n* = 3) assays. A one-way ANOVA was performed on the data sets to obtain the requisite *P* values.

## Additional files

Additional file 1: Table S2. The four distinct sets of genes identified from the various analyses that were performed of differentially expressed genes. Additional file 2: Table S3. Data on copy number alterations compared with the genes found to be differentially expressed between MPNSTs and PNFs. 76/121 genes exhibited concordance between copy number changes and the level of expression. Additional file 3: Figure S1. Results of the q-PCR analysis in relation to the top 20 genes differentially expressed between MPNSTs and PNFs. Additional file 4: Table S1. Primers used for q-PCR analysis and results of the q-PCR analysis.

## Abbreviations

CNA: copy number alteration
DABG: detection above background
HMM: hidden Markov model
MPNST: malignant peripheral nerve sheath tumour
NF1: neurofibromatosis type 1
PNF: peripheral neurofibroma
shRNA: short hairpin RNA

## References

[1] Huson S, Kaufmann D (2008). The neurofibromatoses: classification, clinical features and genetic counselling. Neurofibromatoses (monographs in human genetics), vol. 16.

[2] Upadhyaya M (2011). Genetic basis of tumorigenesis in NF1 malignant peripheral nerve sheath tumors. Front Biosci..

[3] Ferner RE, Gutmann DH. International consensus statement on malignant peripheral nerve sheath tumours in neurofibromatosis 1. Cancer Res. 2002;1(62(5)):1573–7. 11894862

[4] Evans DG, Baser ME, McGaughran J, Sharif S, Howard E, Moran A (2002). Malignant peripheral nerve sheath tumours in neurofibromatosis 1. J Med Genet..

[5] Katz D, Lazar A, Lev D (2009). Malignant peripheral nerve sheath tumour (MPNST): the clinical implications of cellular signalling pathways. Expert Rev Mol Med..

[6] Tucker T, Wolkenstein P, Revuz J, Zeller J, Friedman J (2005). Association between benign and malignant peripheral nerve sheath tumors in NF1. Neurology..

[7] Mautner VF, Kluwe L, Friedrich RE (2010). Clinical characterisation of 29 neurofibromatosis type-1 patients with molecularly ascertained 1.4 Mb type-1 NF1 deletions. J Med Genet..

[8] Ferner RE, Hughes RA, Hall SM, Upadhyaya M, Johnson MR (2004). Neurofibromatous neuropathy in neurofibromatosis 1 (NF1). J Med Genet..

[9] Evans DG, Huson SM, Birch JM (2012). Malignant peripheral nerve sheath tumours in inherited disease. Clin Sarcoma Res..

[10] Beert E, Brems H, Daniëls B (2011). Atypical neurofibromas in neurofibromatosis type 1 are premalignant tumors. Genes Chrom Cancer..

[11] Lothe RA, Karhu R, Mandahl N (1996). Gain of 17q24-qter detected by comparative genomic hybridization in malignant tumors from patients with von Recklinghausen’s neurofibromatosis. Cancer Res..

[12] Bridge RS, Bridge JA, Neff JR, Naumann S, Althof P, Bruch LA (2004). Recurrent chromosomal imbalances and structurally abnormal breakpoints within complex karyotypes of malignant peripheral nerve sheath tumour and malignant triton tumour: a cytogenetic and molecular cytogenetic study. J Clin Pathol..

[13] Watson MA, Perry A, Tihan T (2004). Gene expression profiling reveals unique molecular subtypes of neurofibromatosis type I-associated and sporadic malignant peripheral nerve sheath tumors. Brain Pathol..

[14] Storlazzi CT, Brekke HR, Mandahl N (2006). Identification of a novel amplicon at distal 17q containing the BIRC5/SURVIVIN gene in malignant peripheral nerve sheath tumours. J Pathol..

[15] Shen MH, Mantripragada K, Dumanski JP, Frayling I, Upadhyaya M (2007). Detection of copy number changes at the NF1 locus with improved high-resolution array CGH. Clin Genet..

[16] Kresse SH, Skårn M, Ohnstad HO (2008). DNA copy number changes in high-grade malignant peripheral nerve sheath tumors by array CGH. Mol Cancer..

[17] Mantripragada KK, Spurlock G, Kluwe L (2008). High-resolution DNA copy number profiling of malignant peripheral nerve sheath tumors using targeted microarray-based comparative genomic hybridization. Clin Cancer Res..

[18] Mantripragada KK, Díaz de Ståhl T, Patridge C (2009). Genome-wide high-resolution analysis of DNA copy number alterations in NF1-associated malignant peripheral nerve sheath tumors using 32 K BAC array. Genes Chrom Cancer.

[19] Brekke HR, Kolberg M, Skotheim RI (2009). Identification of p53 as a strong predictor of survival for patients with malignant peripheral nerve sheath tumors. NeuroOncol..

[20] Miller SJ, Jessen WJ, Mehta T (2009). Integrative genomic analyses of neurofibromatosis tumours identify *SOX9* as a biomarker and survival gene. EMBO Mol Med..

[21] Pemov A, Park C, Reilly KM, Stewart DR (2010). Evidence of perturbations of cell cycle and DNA repair pathways as a consequence of human and murine NF1-haploinsufficiency. BMC Genomics..

[22] Brekke HR, Ribeiro FR, Kolberg M (2010). Genomic changes in chromosomes 10, 16, and X in malignant peripheral nerve sheath tumors identify a high-risk patient group. J Clin Oncol..

[23] Subramanian S, Thayanithy V, West RB (2010). Genome-wide transcriptome analyses reveal p53 inactivation mediated loss of miR-34a expression in malignant peripheral nerve sheath tumours. J Pathol..

[24] Chai G, Liu N, Ma J (2010). MicroRNA-10b regulates tumorigenesis in neurofibromatosis type 1. Cancer Sci..

[25] Upadhyaya M, Spurlock G, Thomas L (2012). Microarray-based copy number analysis of neurofibromatosis type-1 (NF1)-associated malignant peripheral nerve sheath tumors reveals a role for Rho-GTPase pathway genes in NF1 tumorigenesis. Hum Mutat..

[26] Mo W, Chen J, Patel A (2013). CXCR4/CXCL12 mediate autocrine cell-cycle progression in NF1-associated malignant peripheral nerve sheath tumors. Cell..

[27] Rahrmann EP, Watson AL, Keng VW (2013). Forward genetic screen for malignant peripheral nerve sheath tumor formation identifies new genes and pathways driving tumorigenesis. Nat Genet..

[28] Watson AL, Rahrmann EP, Moriarity BS (2013). Canonical Wnt/β-catenin signaling drives human Schwann cell transformation, progression, and tumor maintenance. Cancer Discov..

[29] Largaespada D, Ratner N (2013). Interweaving the strands: β-catenin, an HIV co-receptor, and Schwann cell tumors. Cancer Cell..

[30] Luscan A, Shackleford G, Masliah-Planchon J (2013). The activation of the WNTWnt signalling pathway is a hallmark in Neurofibromatosis type 1 tumorigenesis. Clin Cancer Res.

[31] Huang N, Shah PK, Li C (2012). Lessons from a decade of integrating cancer copy number alterations with gene expression profiles. Brief Bioinformatics..

[32] Hassan A, Mokhtar NM, Sin K, Mohammed Rose I, Sagap I, Harun R (2014). Integrated analysis of copy number variation and genome-wide expression profiling in colorectal cancer tissues. PLoS ONE.

[33] Rittling SR, Chambers AF (2004). Role of osteopontin in tumour progression. Brit J Cancer..

[34] Pradervand S, Paillusson A, Thomas J (2008). Affymetrix Whole-Transcript Human Gene1.0 ST array is highly concordant with standard 3′ expression arrays. Biotechniques..

[35] Xi L, Feber A, Gupta V (2008). Whole genome exon arrays identify differential expression of alternatively spliced, cancer-related genes in lung cancer. Nucleic Acids Res..

[36] Thorsen K, Sørensen KD, Brems-Eskildsen AS (2008). Alternative splicing in colon, bladder, and prostate cancer identified by exon array analysis. Mol Cell Proteomics..

[37] Kapur K, Xing Y, Ouyang Z, Wong WH (2007). Exon arrays provide accurate assessments of gene expression. Genome Biol..

[38] Furger KA, Menon RK, Tuck AB, Bramwell VH, Chambers AF (2001). The functional and clinical roles of osteopontin in cancer and metastasis. Current Mol Med..

[39] Bramwell VHC, Tuck AB, Wilson SM (2005). Expression of osteopontin and HGF/met in adult soft tissue tumors. Cancer Biol Ther..

[40] Hoshi N, Sugino T, Suzuki T (2005). Regular expression of osteopontin in granular cell tumor: distinctive feature among Schwannian cell tumors. Pathol Int..

[41] Rangaswami H, Bulbule A, Kundu G (2006). Osteopontin: role in cell signaling and cancer progression. Trends Cell Biol..

[42] Wai PY, Kuo PC (2008). Osteopontin: regulation in tumor metastasis. Cancer Metastasis Rev..

[43] Weber GF (2001). The metastasis gene osteopontin: a candidate target for cancer therapy. Biochim Biophys Acta..

[44] Shevde LA, Das S, Clark DW, Samant RS (2010). Osteopontin: an effector and an effect of tumor metastasis. Curr Mol Med..

[45] Ahmed M, Behera R, Chakraborty G (2011). Osteopontin: a potentially important therapeutic target in cancer. Expert Opin Ther Targets..

[46] Luo X, Ruhland MK, Pazolli E, Lind AC, Stewart SA (2011). Osteopontin stimulates preneoplastic cellular proliferation through activation of the MAPK pathway. Mol Cancer Res..

[47] Rao G, Du L, Chen Q (2013). Osteopontin, a possible modulator of cancer stem cells and their malignant niche. Oncoimmunology..

[48] El-Tanani MK, Campbell FC, Kurisetty V, Jin D, McCann M, Rudland PS (2006). The regulation and role of osteopontin in malignant transformation and cancer. Cytokine Growth Factor Rev..

[49] Tilli TM, Mello KD, Ferreira LB (2012). Both osteopontin-c and osteopontin-b splicing isoforms exert pro-tumorigenic roles in prostate cancer cells. Prostate..

[50] Guo X, Zhang YP, Mitchell DA, Denhardt DT, Chambers AF (1995). Identification of a Ras-activated enhancer in the mouse osteopontin promoter and its interaction with a putative ETS-related transcription factor whose activity correlates with the metastatic potential of the cell. Mol Cell Biol..

[51] Mason CK, McFarlane S, Johnston PG (2008). Agelastatin A: a novel inhibitor of osteopontin-mediated adhesion, invasion, and colony formation. Mol Cancer Ther.

[52] The cancer genome atlas [http://cancergenome.nih.gov]

[53] Gu W, Choi H, Ghosh D (2008). Global associations between copy number and transcript mRNA microarray data: an empirical study. Cancer Inform..

[54] Thomas L, Mautner VF, Cooper DN, Upadhyaya M (2012). Molecular heterogeneity in malignant peripheral nerve sheath tumors associated with neurofibromatosis type 1. Hum Genomics.

[55] Spivey TL, De Giorgi V, Zhao Y (2012). The stable traits of melanoma genetics: an alternate approach to target discovery. BMC Genomics..

[56] Lu TP, Lai LC, Tsai MH (2011). Integrated analyses of copy number variations and gene expression in lung adenocarcinoma. PLoS ONE.

[57] Conan copy number analysis [http://www.sanger.ac.uk/research/projects/cancergenome/copy_number.html]

[58] COSMIC catalogue of somatic mutations in cancer [http://cancer.sanger.ac.uk/cancergenome/projects/cosmic]

[59] Kleinjan DA, Lettice LA (2008). Long-range gene control and genetic disease. Adv Genet..

[60] Henrichsen CN, Chaignat E, Reymond A (2009). Copy number variants, diseases and gene expression. Hum Mol Genet.

[61] Hyman E, Kauraniemi P, Hautaniemi S (2002). Impact of DNA amplification on gene expression patterns in breast cancer. Cancer Res..

[62] White BD, Chien AJ, Dawson DW (2012). Dysregulation of Wnt/β-catenin signaling in gastrointestinal cancers. Gastroenterology..

[63] Reilly KM (2013). Extending the convergence of canonical Wnt signaling and classic cancer pathways for treatment of malignant peripheral nerve sheath tumors. Cancer Discov..

[64] Vinas JL, Sola A, Jung M, Mastora C, Vinuesa E, Pi F (2010). Inhibitory action of Wnt target gene osteopontin on mitochondrial cytochrome c release determines renal ischemic resistance. Am J Physiol Renal Physiol..

[65] Blair SL, Heerdt P, Sachar S (1997). Glutathione metabolism in patients with non-small cell lung cancers. Cancer Res..

[66] Sun Z, Chen J, Aakre J (2011). Genetic variation in glutathione metabolism and DNA repair genes predicts survival of small-cell lung cancer patients predicts survival of small-cell lung cancer patients. Ann Oncol..

[67] Harder A, Titze S, Herbst L, Harder T, Guse K, Tinschert S (2010). Monozygotic twins with neurofibromatosis type 1 (NF1) display differences in methylation of NF1 gene promoter elements, 5′ untranslated region, exon and intron 1. Twin Res Hum Genet.

[68] Titze S, Peters H, Währisch S, Harder T, Guse K, Buske A (2010). Differential MSH2 promoter methylation in blood cells of neurofibromatosis type 1 (NF1) patients. Eur J Hum Genet.

[69] Rohde F, Rimcus C, Friederichs J (2007). Expression of osteopontin, a target gene of de-regulated Wnt signalling predicts survival of colon cancer. Int J Cancer..

[70] Irizarry RA, Hobbs B, Collin F (2003). Exploration, normalization, and summaries of high density oligonucleotide array probe level data. Biostatistics..

