# Supplementary material for: Evaluation of copy number variation and gene expression in neurofibromatosis type-1-associated malignant peripheral nerve sheath tumours
Source: Hum Genomics. 2015 Feb 15;9(1):3. doi: 10.1186/s40246-015-0025-3 (PMC4367978; doi:10.1186/s40246-015-0025-3)
Supplement: Additional file 1: Table S2. — The four distinct sets of genes identified from the various analyses that were performed of differentially expressed genes. [file 40246_2015_25_MOESM1_ESM.pdf]

Additional file 1: Table S2: The 4 distinct sets of genes identified from the various analyses of differentially expressed genes that were performed

	**Full Normalised List**	**Less Stringent Gene List**	**Stringent Gene List**
4 benign vs. 5 malignant	17881	1957	258
3 benign vs. 3 malignant	17881	1259	127
Cross-comparison (common lists)	NA	649	49
