# Supplementary material for: Evaluation of copy number variation and gene expression in neurofibromatosis type-1-associated malignant peripheral nerve sheath tumours
Source: Hum Genomics. 2015 Feb 15;9(1):3. doi: 10.1186/s40246-015-0025-3 (PMC4367978; doi:10.1186/s40246-015-0025-3)
Supplement: Additional file 2: Table S3. — Data on copy number alterations compared with the genes found to be differentially expressed between MPNSTs and PNFs. 76/121 genes exhibited concordance between copy number changes and the level of expression. [file 40246_2015_25_MOESM2_ESM.pdf]

**Additional file 2: Table S3:** Data on copy number alterations with the data on genes found to be differentially expressed between MPNSTs and PNFs. 76/121 genes exhibited concordance between copy number changes and expression analysis

| Comparison Results | | | | | Exon | | | | | HMM | | | Segmentation | | |
|---|---|---|---|---|---|---|---|---|---|---|---|---|---|---|---|
| EntrezGeneID | RefSeqID | Gene | Copy Number Method | Direction results | Transcript ID | Fold-Change Benign vs. Malignant (4v5) | p-value Benign vs. Malignant (4v5) | Fold-Change Benign vs. Malignant (3v3) | p-value Benign vs. Malignant (3v3) | Sample ID | # of Sample Overlap | CNV Type | Sample ID | # of sample overlap | CNV Type |
| 202915 | NM_001097620 | *TMEM184A* | *HMM* | Expression down, copy number up | 3035380 | -1.47 | 0.0020 | -1.32 | 0.0371 | M15T M3T M5T M7T M8T M9T | 6 | Amplification | | | |
| 7274 | NM_000370 | *TTPA* | *HMM & segmentation* | Expression down, copy number up | 3137901 | -1.60 | 0.0093 | -1.35 | 0.0004 | M15T M3T M7T M8T M9T | 5 | Amplification | M15T M3T M7T M8T M9T | 5 | Amplification |
| 5054 | NM_000602 | *SERPINE1* | *HMM & segmentation* | Both up | 3016148 | 4.54 | 0.0008 | 5.74 | 0.0082 | M15T M3T M5T M7T M8T | 5 | Amplification | M15T M2T M3T M5T M7T | 5 | Amplification |
| 6241 | NM_001034 | *RRM2* | *HMM & segmentation* | Expression down, copy number up | 4006326 | -2.65 | 0.0000 | -2.67 | 0.0005 | M15T M2T M3T M7T M8T | 5 | Amplification | M14T M15T M2T M3T M7T M8T | 6 | Amplification |
| 54545 | NM_001040446 | *MTMR12* | *HMM & segmentation* | Expression down, copy number up | 2852274 | -1.52 | 0.0079 | -1.56 | 0.0405 | M15T M2T M3T M7T M8T | 5 | Amplification | M14T M15T M2T M3T M7T M8T | 6 | Amplification |
| 56164 | NM_001122833 | *STK31* | *HMM & segmentation* | Expression down, copy number up | 2993029 | -1.40 | 0.0010 | -1.36 | 0.0287 | M15T M3T M5T M7T M9T | 5 | Amplification | M15T M2T M3T M5T M7T M8T M9T | 7 | Amplification |
| 23213 | NM_001128204 | *SULF1* | *HMM & segmentation* | Both up | 3102372 | 5.70 | 0.0033 | 7.24 | 0.0252 | M15T M3T M7T M8T M9T | 5 | Amplification | M15T M3T M7T M8T M9T | 5 | Amplification |
| 5118 | NM_002593 | *PCOLCE* | *HMM & segmentation* | Both up | 3015682 | 3.83 | 0.0012 | 3.60 | 0.0314 | M15T M3T M5T M7T M8T | 5 | Amplification | M15T M2T M3T M5T M7T M8T | 6 | Amplification |
| 5150 | NM_002603 | *PDE7A* | *HMM & segmentation* | Both up | 3138464 | 2.27 | 0.0014 | 2.25 | 0.0120 | M15T M3T M7T M8T M9T | 5 | Amplification | M15T M3T M7T M8T M9T | 5 | Amplification |
| 5861 | NM_004161 | *RAB1A* | *HMM & segmentation* | Both up | 2556667 | 1.62 | 0.0134 | 1.48 | 0.0166 | M15T M2T M3T M7T M8T | 5 | Amplification | M14T M15T M2T M3T M7T M8T | 6 | Amplification |
| 9242 | NM_005098 | *MSC* | *HMM & segmentation* | Both up | 3140213 | 3.25 | 0.0012 | 2.44 | 0.0243 | M15T M3T M7T M8T M9T | 5 | Amplification | M15T M3T M7T M8T M9T | 5 | Amplification |
| 1956 | NM_005228 | *EGFR* | *HMM & segmentation* | Expression down, copy number up | 3002640 | -2.47 | 0.0114 | -3.29 | 0.0231 | M15T M3T M5T M8T M9T | 5 | Amplification | M15T M2T M3T M5T M8T M9T | 6 | Amplification |
| 6921 | NM_005648 | *TCEB1* | *HMM & segmentation* | Expression down, copy number up | 3590498 | -1.60 | 0.0157 | -1.71 | 0.0494 | M15T M3T M7T M8T M9T | 5 | Amplification | M15T M3T M7T M8T M9T | 5 | Amplification |
| 22948 | NM_012073 | *CCT5* | *HMM & segmentation* | Both up | 2801526 | 1.82 | 0.0079 | 1.64 | 0.0006 | M15T M2T M3T M7T M8T | 5 | Amplification | M14T M15T M2T M3T M7T M8T | 6 | Amplification |
| 9771 | NM_012294 | *RAPGEF5* | *HMM & segmentation* | Expression down, copy number up | 3040967 | -4.25 | 0.0045 | -3.41 | 0.0056 | M15T M3T M5T M7T M8T M9T | 6 | Amplification | M15T M2T M3T M5T M7T M8T M9T | 7 | Amplification |
| 26608 | NM_012453 | *TBL2* | *HMM & segmentation* | Both up | 3056131 | 1.32 | 0.0058 | 1.38 | 0.0123 | M15T M3T M5T M7T M8T | 5 | Amplification | M15T M2T M3T M5T M7T M8T | 6 | Amplification |
| 9760 | NM_014729 | *TOX* | *HMM & segmentation* | Expression down, copy number up | 3136888 | -4.85 | 0.0059 | -6.07 | 0.0213 | M15T M3T M7T M8T M9T | 5 | Amplification | M15T M3T M7T M8T M9T | 5 | Amplification |
| 22848 | NM_014911 | *AAK1* | *HMM & segmentation* | Both up | 2558150 | 1.76 | 0.0107 | 1.80 | 0.0169 | M15T M2T M3T M7T M8T | 5 | Amplification | M14T M15T M2T M3T M7T M8T | 6 | Amplification |
| 25928 | NM_015464 | *SOSTDC1* | *HMM & segmentation* | Expression down, copy number up | 3039671 | -2.35 | 0.0239 | -2.10 | 0.0142 | M15T M3T M5T M7T M8T M9T | 6 | Amplification | M15T M2T M3T M5T M7T M8T M9T | 7 | Amplification |
| 51373 | NM_015969 | *MRPS17* | *HMM & segmentation* | Expression down, copy number up | 3003107 | -1.95 | 0.0045 | -1.99 | 0.0390 | M15T M3T M5T M8T M9T | 5 | Amplification | M15T M2T M3T M5T M8T M9T | 6 | Amplification |
| 55704 | NM_018084 | *CCDC88A* | *HMM & segmentation* | Both up | 2553771 | 2.64 | 0.0045 | 3.12 | 0.0074 | M15T M2T M3T M7T M8T | 5 | Amplification | M14T M15T M2T M3T M7T M8T | 6 | Amplification |
| 79888 | NM_024830 | *LPCAT1* | *HMM & segmentation* | Both up | 2845973 | 1.67 | 0.0069 | 1.65 | 0.0284 | M15T M2T M3T M7T M8T | 5 | Amplification | M14T M15T M2T M3T M7T M8T | 6 | Amplification |
| 80727 | NM_025250 | *TTYH3* | *HMM & segmentation* | Both up | 2987632 | 2.14 | 0.0009 | 2.53 | 0.0019 | M15T M3T M5T M7T M8T M9T | 6 | Amplification | M15T M2T M3T M5T M7T M8T M9T | 7 | Amplification |
| 286183 | NM_173688 | *NKAIN3* | *HMM & segmentation* | Expression down, copy number up | 3100690 | -2.33 | 0.0033 | -2.46 | 0.0261 | M15T M3T M7T M8T M9T | 5 | Amplification | M15T M3T M7T M8T M9T | 5 | Amplification |
| 221823 | NM_175886 | *PRPS1L1* | *HMM & segmentation* | Expression down, copy number up | 3040175 | -1.30 | 0.0087 | -1.30 | 0.0070 | M15T M3T M5T M7T M8T M9T | 6 | Amplification | M15T M2T M3T M5T M7T M8T M9T | 7 | Amplification |
| 349075 | NM_182633 | *ZNF713* | *HMM & segmentation* | Expression down, copy number up | 3003107 | -1.95 | 0.0045 | -1.99 | 0.0390 | M15T M3T M5T M8T M9T | 5 | Amplification | M15T M2T M3T M5T M8T M9T | 6 | Amplification |
| 54476 | NM_207111 | *RNF216* | *HMM & segmentation* | Both up | 3036985 | 1.52 | 0.0151 | 1.68 | 0.0007 | M15T M3T M5T M7T M8T M9T | 6 | Amplification | M15T M2T M3T M5T M7T M8T M9T | 7 | Amplification |
| 3691 | NM_000213 | *ITGB4* | *segmentation* | Expression down, copy number up | 3735151 | -3.58 | 0.0031 | -3.96 | 0.0051 | | | | M15T M2T M3T M5T M8T | 5 | Amplification |
| 3559 | NM_000417 | *IL2RA* | *segmentation* | Expression up, Copy number down | 3275729 | 4.04 | 0.0031 | 5.03 | 0.0174 | | | | M15T M3T M5T M7T M8T | 5 | Deletion |
| 2944 | NM_000561 | *GSTM1* | *segmentation* | Both down | 2350952 | -2.27 | 0.0266 | -3.25 | 0.0177 | | | | M15T M2T M5T M7T M8T | 5 | Deletion |
| 3082 | NM_000601 | *HGF* | *segmentation* | Both up | 3058944 | 10.97 | 0.0010 | 7.80 | 0.0170 | | | | M15T M2T M3T M5T M7T | 5 | Amplification |
| 596 | NM_000633 | *BCL2* | *segmentation* | Expression down, copy number up | 3811339 | -2.63 | 0.0018 | -2.49 | 0.0287 | | | | M14T M3T M7T M8T M9T | 5 | Amplification |
| 2246 | NM_000800 | *FGF1* | *segmentation* | Both down | 2879166 | -3.76 | 0.0025 | -3.24 | 0.0406 | | | | M14T M15T M2T M7T M8T | 5 | Deletion |
| 2946 | NM_000848 | *GSTM2* | *segmentation* | Both down | 2350952 | -2.27 | 0.0266 | -3.25 | 0.0177 | | | | M15T M2T M3T M5T M7T M8T | 6 | Deletion |
| 2948 | NM_000850 | *GSTM4* | *segmentation* | Both down | 2350952 | -2.27 | 0.0266 | -3.25 | 0.0177 | | | | M15T M2T M3T M5T M7T M8T | 6 | Deletion |
| 51155 | NM_001002032 | *HN1* | *segmentation* | Both up | 3770606 | 2.85 | 0.0011 | 2.69 | 0.0009 | | | | M15T M2T M3T M5T M8T | 5 | Amplification |
| 3983 | NM_001003407 | *ABLIM1* | *segmentation* | Both down | 3307939 | -2.50 | 0.0069 | -3.03 | 0.0177 | | | | M15T M3T M5T M7T M8T | 5 | Deletion |
| 2123 | NM_001003927 | *EVI2A* | *segmentation* | Expression up, Copy number down | 3752271 | 2.99 | 0.0192 | 3.19 | 0.0340 | | | | M15T M2T M3T M5T M7T M8T | 6 | Deletion |
| 9184 | NM_001007793 | *BUB3* | *segmentation* | Expression up, Copy number down | 3268669 | 1.61 | 0.0074 | 1.62 | 0.0335 | | | | M15T M3T M5T M7T M8T | 5 | Deletion |
| 4601 | NM_001008541 | *MXI1* | *segmentation* | Both down | 3263624 | -1.79 | 0.0036 | -1.84 | 0.0286 | | | | M15T M3T M5T M7T M8T | 5 | Deletion |
| 286187 | NM_001013626 | *LRRC67* | *segmentation* | Expression down, copy number up | 3138929 | -1.33 | 0.0091 | -1.36 | 0.0084 | | | | M15T M3T M7T M8T M9T | 5 | Amplification |
| 6224 | NM_001023 | *RPS20* | *segmentation* | Expression down, copy number up | 3136129 | -1.34 | 0.0036 | -1.36 | 0.0326 | | | | M15T M3T M7T M8T M9T | 5 | Amplification |
| 2976 | NM_001035521 | *GTF3C2* | *segmentation* | Both up | 2545681 | 1.68 | 0.0019 | 1.55 | 0.0173 | | | | M14T M2T M3T M7T M8T | 5 | Amplification |
| 9123 | NM_001042422 | *SLC16A3* | *segmentation* | Both up | 3738629 | 1.99 | 0.0065 | 2.42 | 0.0120 | | | | M15T M2T M3T M5T M8T | 5 | Amplification |
| 7412 | NM_001078 | *VCAM1* | *segmentation* | Expression up, Copy number down | 2348992 | 6.21 | 0.0037 | 5.06 | 0.0376 | | | | M15T M2T M3T M5T M7T M8T | 6 | Deletion |
| 729330 | NM_001080399 | *OC90* | *segmentation* | Expression down, copy number up | 3153961 | -1.42 | 0.0005 | -1.33 | 0.0022 | | | | M15T M3T M7T M8T M9T | 5 | Amplification |
| 401474 | NM_001101676 | *SAMD12* | *segmentation* | Expression down, copy number up | 3150289 | -4.38 | 0.0021 | -4.93 | 0.0023 | | | | M15T M3T M7T M8T M9T | 5 | Amplification |
| 1075 | NM_001114173 | *CTSC* | *segmentation* | Expression up, Copy number down | 3385769 | 2.29 | 0.0096 | 2.39 | 0.0207 | | | | M15T M2T M3T M7T M8T | 5 | Deletion |
| 57157 | NM_001127357 | *PHTF2* | *segmentation* | Both up | 3010082 | 2.01 | 0.0000 | 1.97 | 0.0042 | | | | M15T M2T M3T M5T M7T | 5 | Amplification |
| 1601 | NM_001343 | *DAB2* | *segmentation* | Both up | 2854445 | 2.65 | 0.0278 | 3.46 | 0.0352 | | | | M14T M15T M2T M3T M9T | 5 | Amplification |
| 2587 | NM_001480 | *GALR1* | *segmentation* | Expression down, copy number up | 3794641 | -1.92 | 0.0016 | -1.92 | 0.0321 | | | | M14T M15T M3T M7T M8T M9T | 6 | Amplification |
| 3631 | NM_001566 | *INPP4A* | *segmentation* | Both up | 2495446 | 1.76 | 0.0010 | 1.90 | 0.0038 | | | | M14T M2T M3T M7T M8T | 5 | Amplification |
| 1453 | NM_001893 | *CSNK1D* | *segmentation* | Both up | 3738629 | 1.99 | 0.0065 | 2.42 | 0.0120 | | | | M15T M2T M3T M5T M8T | 5 | Amplification |
| 3575 | NM_002185 | *IL7R* | *segmentation* | Both up | 2806468 | 4.78 | 0.0034 | 4.11 | 0.0288 | | | | M14T M15T M2T M3T M7T M8T M9T | 7 | Amplification |
| 3984 | NM_002314 | *LIMK1* | *segmentation* | Both up | 3008108 | 2.23 | 0.0015 | 2.04 | 0.0341 | | | | M15T M2T M3T M5T M7T | 5 | Amplification |
| 4288 | NM_002417 | *MKI67* | *segmentation* | Expression up, Copy number down | 3312490 | 3.84 | 0.0401 | 4.54 | 0.0204 | | | | M15T M3T M5T M7T M8T | 5 | Deletion |
| 5862 | NM_002865 | *RAB2A* | *segmentation* | Both up | 3100166 | 2.01 | 0.0023 | 1.71 | 0.0488 | | | | M15T M3T M7T M8T M9T | 5 | Amplification |
| 6427 | NM_003016 | *SFRS2* | *segmentation* | Both up | 3771800 | 1.48 | 0.0320 | 1.73 | 0.0012 | | | | M15T M2T M3T M5T M8T | 5 | Amplification |
| 6674 | NM_003114 | *SPAG1* | *segmentation* | Expression down, copy number up | 3109201 | -1.64 | 0.0014 | -1.54 | 0.0188 | | | | M15T M3T M7T M8T M9T | 5 | Amplification |
| 7514 | NM_003400 | *XPO1* | *segmentation* | Both up | 2555490 | 2.01 | 0.0060 | 1.81 | 0.0499 | | | | M14T M15T M2T M3T M7T M8T | 6 | Amplification |
| 403 | NM_004311 | *ARL3* | *segmentation* | Both down | 3304475 | -1.77 | 0.0080 | -1.83 | 0.0047 | | | | M15T M3T M5T M7T M8T | 5 | Deletion |
| 4225 | NM_005925 | *MEP1B* | *segmentation* | Both down | 3783788 | -1.48 | 0.0027 | -1.53 | 0.0096 | | | | M15T M2T M5T M7T M8T | 5 | Deletion |
| 7444 | NM_006296 | *VRK2* | *segmentation* | Both up | 2483451 | 1.66 | 0.0129 | 1.75 | 0.0301 | | | | M14T M15T M2T M3T M7T M8T | 6 | Amplification |
| 8100 | NM_006531 | *IFT88* | *segmentation* | Both down | 3480411 | -1.71 | 0.0040 | -1.74 | 0.0498 | | | | M2T M3T M5T M7T M8T | 5 | Deletion |
| 10771 | NM_006624 | *ZMYND11* | *segmentation* | Both down | 3231389 | -1.65 | 0.0050 | -1.94 | 0.0043 | | | | M15T M3T M5T M7T M8T | 5 | Deletion |
| 11185 | NM_006774 | *INMT* | *segmentation* | Expression down, copy number up | 2995476 | -2.18 | 0.0080 | -2.28 | 0.0246 | | | | M15T M2T M3T M5T M8T M9T | 6 | Amplification |
| 10966 | NM_006822 | *RAB40B* | *segmentation* | Expression down, copy number up | 3775211 | -1.90 | 0.0006 | -1.75 | 0.0098 | | | | M15T M2T M3T M5T M8T | 5 | Amplification |
| 11036 | NM_006872 | *GTF2A1L* | *segmentation* | Expression down, copy number up | 2481379 | -1.83 | 0.0327 | -2.17 | 0.0453 | | | | M14T M15T M2T M3T M7T M8T | 6 | Amplification |
| 11037 | NM_006873 | *STON1* | *segmentation* | Expression down, copy number up | 2481379 | -1.83 | 0.0327 | -2.17 | 0.0453 | | | | M14T M15T M2T M3T M7T M8T | 6 | Amplification |

| | | | | | | | | | | | | | | | |
|---|---|---|---|---|---|---|---|---|---|---|---|---|---|---|---|
| 11196 | NM_007190 | *SEC23IP* | *segmentation* | Expression up, Copy number down | 3267455 | 1.77 | 0.0032 | 1.83 | 0.0292 | | | | M15T M3T M5T M7T M8T | 5 | Deletion |
| 11330 | NM_007272 | *CTRC* | *segmentation* | Both down | 2321797 | -1.74 | 0.0075 | -1.43 | 0.0211 | | | | M15T M2T M5T M7T M8T | 5 | Deletion |
| 23530 | NM_012343 | *NNT* | *segmentation* | Both up | 2808438 | 2.17 | 0.0273 | 2.02 | 0.0151 | | | | M14T M15T M2T M3T M9T | 5 | Amplification |
| 23639 | NM_012472 | *LRRC6* | *segmentation* | Expression down, copy number up | 3154136 | -3.74 | 0.0022 | -2.36 | 0.0151 | | | | M15T M3T M7T M8T M9T | 5 | Amplification |
| 10113 | NM_013388 | *PREB* | *segmentation* | Both up | 2545509 | 1.43 | 0.0278 | 1.45 | 0.0249 | | | | M14T M2T M3T M7T M8T | 5 | Amplification |
| 27044 | NM_014390 | *SND1* | *segmentation* | Both up | 3022465 | 1.77 | 0.0094 | 1.71 | 0.0334 | | | | M15T M2T M3T M5T M7T | 5 | Amplification |
| 9913 | NM_014860 | *SUPT7L* | *segmentation* | Both up | 2546008 | 1.84 | 0.0113 | 1.79 | 0.0271 | | | | M14T M2T M3T M7T M8T | 5 | Amplification |
| 22876 | NM_014937 | *INPP5F* | *segmentation* | Both down | 3267382 | -4.57 | 0.0003 | -4.40 | 0.0065 | | | | M15T M3T M5T M7T M8T | 5 | Deletion |
| 22979 | NM_014971 | *EFR3B* | *segmentation* | Expression down, copy number up | 2473376 | -1.47 | 0.0094 | -1.42 | 0.0492 | | | | M14T M2T M3T M7T M8T | 5 | Amplification |
| 23382 | NM_015328 | *AHCYL2* | *segmentation* | Expression down, copy number up | 3023384 | -1.93 | 0.0071 | -2.26 | 0.0231 | | | | M15T M2T M3T M5T M7T | 5 | Amplification |
| 51631 | NM_016019 | *LUC7L2* | *segmentation* | Expression down, copy number up | 3026988 | -1.34 | 0.0441 | -1.49 | 0.0014 | | | | M15T M2T M3T M5T M7T | 5 | Amplification |
| 57448 | NM_016252 | *BIRC6* | *segmentation* | Both up | 2476219 | 1.62 | 0.0385 | 1.50 | 0.0321 | | | | M14T M2T M3T M7T M8T | 5 | Amplification |
| 51571 | NM_016623 | *FAM49B* | *segmentation* | Both up | 3153328 | 1.81 | 0.0375 | 2.21 | 0.0468 | | | | M15T M3T M7T M8T M9T | 5 | Amplification |
| 55654 | NM_017849 | *TMEM127* | *segmentation* | Both up | 2565246 | 2.34 | 0.0052 | 1.73 | 0.0404 | | | | M14T M15T M2T M3T M7T M8T | 6 | Amplification |
| 54970 | NM_017868 | *TTC12* | *segmentation* | Both down | 3349453 | -1.97 | 0.0161 | -1.43 | 0.0027 | | | | M15T M2T M3T M7T M8T | 5 | Deletion |
| 55666 | NM_017921 | *NPLOC4* | *segmentation* | Both up | 3774029 | 1.90 | 0.0173 | 1.79 | 0.0194 | | | | M15T M2T M3T M5T M8T | 5 | Amplification |
| 55676 | NM_017964 | *SLC30A6* | *segmentation* | Both up | 2476116 | 2.26 | 0.0001 | 2.23 | 0.0019 | | | | M14T M2T M3T M7T M8T | 5 | Amplification |
| 50807 | NM_018482 | *DDEF1* | *segmentation* | Both up | 3153428 | 1.80 | 0.0143 | 2.04 | 0.0075 | | | | M15T M3T M7T M8T M9T | 5 | Amplification |
| 54443 | NM_018685 | *ANLN* | *segmentation* | Both up | 2997376 | 4.95 | 0.0311 | 7.53 | 0.0466 | | | | M15T M2T M3T M5T M8T M9T | 6 | Amplification |
| 55924 | NM_019099 | *C1orf183* | *segmentation* | Both down | 2428079 | -1.56 | 0.0143 | -1.41 | 0.0416 | | | | M2T M3T M5T M7T M8T | 5 | Deletion |
| 57835 | NM_021196 | *SLC4A5* | *segmentation* | Expression down, copy number up | 2559849 | -1.64 | 0.0026 | -1.54 | 0.0361 | | | | M14T M15T M2T M3T M7T M8T | 6 | Amplification |
| 60370 | NM_021732 | *AVPI1* | *segmentation* | Both down | 3302495 | -3.82 | 0.0018 | -2.77 | 0.0196 | | | | M15T M3T M5T M7T M8T | 5 | Deletion |
| 64224 | NM_022373 | *HERPUD2* | *segmentation* | Both up | 3045739 | 1.31 | 0.0044 | 1.36 | 0.0226 | | | | M15T M2T M3T M5T M8T M9T | 6 | Amplification |
| 64418 | NM_022484 | *TMEM168* | *segmentation* | Both up | 3068476 | 1.65 | 0.0055 | 1.72 | 0.0465 | | | | M15T M2T M3T M5T M7T | 5 | Amplification |
| 64427 | NM_022492 | *TTC31* | *segmentation* | Both up | 2489322 | 1.39 | 0.0114 | 1.45 | 0.0134 | | | | M14T M15T M2T M3T M7T M8T | 6 | Amplification |
| 64762 | NM_022751 | *FAM59A* | *segmentation* | Both down | 3803290 | -2.81 | 0.0043 | -3.35 | 0.0262 | | | | M15T M2T M5T M7T M8T | 5 | Deletion |
| 64895 | NM_022894 | *PAPOLG* | *segmentation* | Both up | 2484305 | 1.76 | 0.0026 | 2.05 | 0.0041 | | | | M14T M15T M2T M3T M7T M8T | 6 | Amplification |
| 80059 | NM_024993 | *LRRTM4* | *segmentation* | Expression down, copy number up | 2560881 | -7.50 | 0.0009 | -11.89 | 0.0045 | | | | M14T M15T M2T M3T M7T M8T | 6 | Amplification |
| 259217 | NM_025015 | *HSPA12A* | *segmentation* | Both down | 3308397 | -4.93 | 0.0062 | -5.92 | 0.0053 | | | | M15T M3T M5T M7T M8T | 5 | Deletion |
| 80217 | NM_025145 | *C10orf79* | *segmentation* | Both down | 3305198 | -1.96 | 0.0009 | -1.92 | 0.0238 | | | | M15T M3T M5T M7T M8T | 5 | Deletion |
| 80705 | NM_025244 | *TSGA10* | *segmentation* | Expression down, copy number up | 2566586 | -1.74 | 0.0068 | -1.87 | 0.0299 | | | | M14T M2T M3T M7T M8T | 5 | Amplification |
| 81553 | NM_030797 | *FAM49A* | *segmentation* | Both up | 2541699 | 3.22 | 0.0056 | 3.19 | 0.0455 | | | | M14T M2T M3T M7T M8T | 5 | Amplification |
| 83539 | NM_031422 | *CHST9* | *segmentation* | Both down | 3802416 | -3.30 | 0.0106 | -2.77 | 0.0091 | | | | M15T M2T M5T M7T M8T | 5 | Deletion |
| 83998 | NM_032044 | *REG4* | *segmentation* | Both down | 2431066 | -1.36 | 0.0003 | -1.36 | 0.0067 | | | | M2T M3T M5T M7T M8T | 5 | Deletion |
| 84912 | NM_032826 | *SLC35B4* | *segmentation* | Both up | 3074039 | 1.97 | 0.0123 | 1.80 | 0.0351 | | | | M15T M2T M3T M5T M7T | 5 | Amplification |
| 84928 | NM_032842 | *TMEM209* | *segmentation* | Both up | 3072435 | 1.33 | 0.0342 | 1.49 | 0.0326 | | | | M15T M2T M3T M5T M7T | 5 | Amplification |
| 90827 | NM_033273 | *ZNF479* | *segmentation* | Expression down, copy number up | 3004009 | -1.48 | 0.0028 | -1.40 | 0.0483 | | | | M15T M2T M3T M5T M8T M9T | 6 | Amplification |
| 118663 | NM_144587 | *BTBD16* | *segmentation* | Both down | 3268222 | -1.55 | 0.0069 | -1.31 | 0.0065 | | | | M15T M3T M5T M7T M8T | 5 | Deletion |
| 220136 | NM_145020 | *CCDC11* | *segmentation* | Both down | 3807732 | -1.61 | 0.0015 | -1.66 | 0.0038 | | | | M15T M2T M5T M7T M8T | 5 | Deletion |
| 143879 | NM_152433 | *KBTBD3* | *segmentation* | Both down | 3389566 | -1.94 | 0.0219 | -1.64 | 0.0029 | | | | M15T M2T M3T M7T M8T | 5 | Deletion |
| 222166 | NM_152793 | *C7orf41* | *segmentation* | Expression down, copy number up | 2995254 | -4.70 | 0.0083 | -8.03 | 0.0111 | | | | M15T M2T M3T M5T M8T M9T | 6 | Amplification |
| 151056 | NM_153021 | *PLB1* | *segmentation* | Both up | 2475116 | 1.58 | 0.0148 | 1.61 | 0.0369 | | | | M14T M2T M3T M7T M8T | 5 | Amplification |
| 2849 | NM_153442 | *GPR26* | *segmentation* | Both down | 3268895 | -1.51 | 0.0054 | -1.47 | 0.0452 | | | | M15T M3T M5T M7T M8T | 5 | Deletion |
| 286749 | NM_172311 | *STON1-GTF2A1L* | *segmentation* | Expression down, copy number up | 2481379 | -1.83 | 0.0327 | -2.17 | 0.0453 | | | | M14T M15T M2T M3T M7T M8T | 6 | Amplification |
| 257194 | NM_173808 | *NEGR1* | *segmentation* | Both down | 2418078 | -5.20 | 0.0006 | -6.56 | 0.0023 | | | | M15T M2T M3T M5T M8T | 5 | Deletion |
| 220382 | NM_175885 | *MGC33846* | *segmentation* | Both down | 3384248 | -1.84 | 0.0000 | -1.61 | 0.0011 | | | | M15T M2T M3T M7T M8T | 5 | Deletion |
| 550 | NM_181575 | *AUP1* | *segmentation* | Both up | 2560254 | 2.09 | 0.0087 | 1.82 | 0.0030 | | | | M14T M15T M2T M3T M7T M8T | 6 | Amplification |
| 157378 | NM_194291 | *TMEM65* | *segmentation* | Both up | 3151883 | 1.69 | 0.0103 | 2.06 | 0.0071 | | | | M15T M3T M7T M8T M9T | 5 | Amplification |
| 200728 | NM_198276 | *TMEM17* | *segmentation* | Expression down, copy number up | 2555830 | -1.86 | 0.0077 | -2.09 | 0.0223 | | | | M14T M15T M2T M3T M7T M8T | 6 | Amplification |
| 340706 | NM_198496 | *VWA2* | *segmentation* | Both down | 3265224 | -1.61 | 0.0003 | -1.57 | 0.0054 | | | | M15T M3T M5T M7T M8T | 5 | Deletion |
| 374355 | NM_198515 | *C10orf96* | *segmentation* | Both down | 3265809 | -1.44 | 0.0081 | -1.38 | 0.0212 | | | | M15T M3T M5T M7T M8T | 5 | Deletion |
| 375611 | NM_198999 | *SLC26A5* | *segmentation* | Expression down, copy number up | 3065684 | -1.89 | 0.0010 | -1.60 | 0.0196 | | | | M15T M2T M3T M5T M7T | 5 | Amplification |
