# Supplementary material for: Evaluation of copy number variation and gene expression in neurofibromatosis type-1-associated malignant peripheral nerve sheath tumours
Source: Hum Genomics. 2015 Feb 15;9(1):3. doi: 10.1186/s40246-015-0025-3 (PMC4367978; doi:10.1186/s40246-015-0025-3)
Supplement: Additional file 3: Figure S1. — Results of the q-PCR analysis in relation to the top 20 genes differentially expressed between MPNSTs and PNFs. [file 40246_2015_25_MOESM3_ESM.docx]

**Additional file 3: Figure S1.** Results of the q-PCR analysis in relation to the top 20 genes differentially expressed between MPNSTs and PNFs
