# Supplementary material for: Evaluation of copy number variation and gene expression in neurofibromatosis type-1-associated malignant peripheral nerve sheath tumours
Source: Hum Genomics. 2015 Feb 15;9(1):3. doi: 10.1186/s40246-015-0025-3 (PMC4367978; doi:10.1186/s40246-015-0025-3)
Supplement: Additional file 4: Table S1. — Primers used for q-PCR analysis and results of the q-PCR analysis. [file 40246_2015_25_MOESM4_ESM.pdf]

Additional file 4: Table S1: Primers used for q-PCR analysis and results of the q-PCR analysis

Gene ID	Forward Primer (5'-3')	Reverse Primer (5'-3')	Size (bp)
ADH1B	AGCCCATTCACCACTTCCT	AGTACACGGTGGTGGATGAG	59
CCT5	CTTTGTGTGTCATCCGGAAC	CTGCTGAGATATCCTGTGCC	77
CD72	AAACTTGGTCATGGTGGACA	ACAGTCCTTTGCACTGAGTTG	114
COL10A1	ATGCTGCCACAAATACCCTT	AAGGCCCACTACCCAACAC	113
CSRP1	TGCTACGGCAAGAAGTATGG	ACCCTCAGCACTGACAAGG	73
EFHC2	TTGACTGTTGGAAACCTTGC	ATTTCTTAATCGCACTGGCC	105
FAM177A1	TCAATTTATGGCCGAGTTTG	TGCCACCATACAGAGTCAGAT	77
FAP	TAAATTAGCATATGTCTATCAAAACAAT	TCAAATAACATTTAATGGAAGAGA	87
FLJ42200/AK124194	CATGAGAAACTCTTTTGAGAA	TTTCAAAAGCTTTGAATATAAAG	74
GRIK2	TTGGTGGCATCTTCATTGTT	TTTGTGGCAGTGGGAGAAT	66
GRIK3	TGGACAACAAGGACACCTTC	CAGTACCTCAAGTGGCGGT	96
GTDC1	TTTCAGCCAGAGCAGAGAGA	CTGCACGACAAGAATATTTGG	100
KIAA0746	TTAACCTGGCCCTGCTAATC	TCTCCATTCTCCAGGAACTGT	114
KRTAP13-4	CTACCCAGGCTCCTACCCC	CGTTCCTCTCTCTACAGGGA	90
NSBP1/HMGN5	GTACAGGTCGTGCTGCAGTT	GAGAGCTACAACAATGCCCA	124
PEG3	ATCTTTCCCTTCCTGTGGTG	ACTCCAGGGCTTATGAGTCC	80
PTGIS	GCCGGCTACCTGACTCTTTA	GATGTCTTCCACACCTTTCG	101
SLC25A12	TTTGGACAGACTATTATTCATCATCA	GCATAACCGGAAGAAGCATC	91
SPP1	AGGACCTGAACGCGCCTTCT	AGCCAATGATGAGAGCAATGAG	143
TDP1	TGGTCAGCTGAGACTTCTGG	TGCTTGGTTCCTTGTCACA	102
